# Cadmium’s silent sabotage: unveiling its impact on antibiotic efficacy

**DOI:** 10.3389/fmicb.2025.1658173

**Published:** 2025-09-03

**Authors:** Xuan Tao, Hong Zhou, Haoda Yu, Yan Wu, Tao Bian

**Affiliations:** Department of Respiratory Medicine, Wuxi Medical Center, Wuxi People's Hospital, The Affiliated Wuxi People's Hospital of Nanjing Medical University, Nanjing Medical University, Wuxi, China

**Keywords:** antibiotic tolerance, cadmium accumulation, bacterial metabolism, gut microbiota, multi-omics analysis

## Abstract

Despite advancements in non-antibiotic therapies, antibiotics continue to be the cornerstone of bacterial infection management. However, the overuse of antibiotics has led to an increase in clinical failures, a situation worsened by the phenomenon of bacterial antibiotic tolerance, which remains less understood than genetic resistance. Environmental stressors, including heavy metals like cadmium, have been associated with heightened susceptibility to infections, yet their influence on antibiotic efficacy has not been thoroughly investigated. In this study, we demonstrate that chronic exposure to cadmium diminishes the effectiveness of antibiotics in systemic infections, as evidenced by a mouse model. From a mechanistic perspective, alterations in the composition of endogenous metabolites due to changes in gut microbiota, notably the diminished production of DL-mevalonolactone, impede bacterial clearance. This is because DL-mevalonolactone plays a crucial role in facilitating the eradication of antibiotic-tolerant bacteria by activating their metabolic processes. Our findings underscore the detrimental impact of cadmium on antibiotic treatment, emphasizing the health risks associated with cadmium exposure.

## 1 Introduction

Despite the advancement of numerous non-antibiotic pharmaceuticals in recent years, antibiotic therapy continues to be the primary approach for managing bacterial infections (Ghosh et al., 2019). The global antibiotic market remains clinically indispensable, with projections estimating its growth to $57.2 billion by 2030, coinciding with escalating resistance rates (Laxminarayan, 2022). This expansion has persisted a 46% increase in global antibiotic consumption since 2000 (Klein et al., 2018), with over half of infections now exhibiting diminished susceptibility to these drugs (Murray et al., 2022). The misuse and overuse of antibiotics have precipitated an increase in clinical failures, posing a significant threat to public health (Bäumler, 2024; Meek et al., 2015). Recent surveillance data indicates that antimicrobial resistance contributes to nearly 5 million deaths annually worldwide (Murray et al., 2022).

Bacteria that are refractory to antibiotics can survive by employing diverse protective mechanisms under antibiotic pressure. These mechanisms can be broadly categorized into genetic factors (Lopatkin et al., 2021; Beggs et al., 2020) that mediate antibiotic resistance and physiological factors (Lee and Collins, 2012; Pontes Mauricio and Groisman Eduardo, 2020; Fridman et al., 2014) that mediate antibiotic tolerance. While resistance mechanisms have been extensively characterized, tolerance phenotypes remain diagnostically challenging to detect and clinically problematic to treat (Balaban et al., 2019). Unlike the well-defined triggers of antibiotic resistance, which involve the acquisition of resistance genes, the physiological factors contributing to antibiotic tolerance are difficult to distinguish and not well understood (Brauner et al., 2016), particularly regarding the influence of environmental stressors. Among various extreme environments, heavy metal stress is relatively common within human physiology (Koyama et al., 2024; Kaur et al., 2021; Bao et al., 2021).

Cadmium’s environmental persistence and potential for bioaccumulation render it a significant global threat, as evidenced by a 50–100% increase in soil concentrations in industrialized regions over the past century (Tóth et al., 2016). This phenomenon is particularly pronounced in agricultural soils, where the application of phosphate fertilizers has substantially contributed to cadmium accumulation (Jiao et al., 2012). Chronic or repeated low-dose exposure to cadmium is significantly associated with the onset and progression of various diseases, including cardiovascular and cerebrovascular conditions (Fagerberg and Barregard, 2021), renal insufficiency (Tsai et al., 2017), cancer (Derkacz et al., 2024), male reproductive dysfunction (Bhardwaj et al., 2024), and decreased bone mineral density (Wallin et al., 2016). Notably, there is an increasing body of evidence suggesting that chronic cadmium exposure is probably linked to heightened susceptibility to and severity of bacterial and viral infections (Liu et al., 2025). The immunomodulatory effects of cadmium exposure may exacerbate complications related to infectious diseases. A single-cell transcriptomic analysis has revealed a marked reduction in the CD14^+^ monocyte subset and a consequent diminished capacity to clear bacterial infections in groups exposed to cadmium (Lu et al., 2021), garnering public attention. This observation is consistent with emerging evidence indicating that heavy metals can disrupt both innate and adaptive immune responses through various pathways (Wang et al., 2021). However, a clinically significant question in the field of infectious diseases—whether chronic cadmium exposure impairs the efficacy of antibiotic treatments—remains unexplored.

This knowledge gap is particularly concerning, as global health assessments reveal widespread cadmium exposure, with significant populations in contaminated regions exceeding safety limits (Rehman et al., 2018). In this study, we demonstrated that chronic cadmium exposure reduces antibiotic efficacy in a mouse model of systemic infections. To assess whether cadmium impairs antibiotic efficacy through gut microbiota-derived metabolites, an in-depth investigation was undertaken to elucidate the underlying mechanisms, focusing on the role of DL-mevalonolactone, which could facilitate the eradication of antibiotic-tolerant bacteria by activating bacterial metabolism. Our findings highlight the potential health risks associated with cadmium bioaccumulation in the human body and inform novel strategies to address antibiotic treatment failures in exposed populations.

## 2 Results

### 2.1 Chronic cadmium exposure results in the less effective bacterial clearance of antibiotics

**Figure 1:**
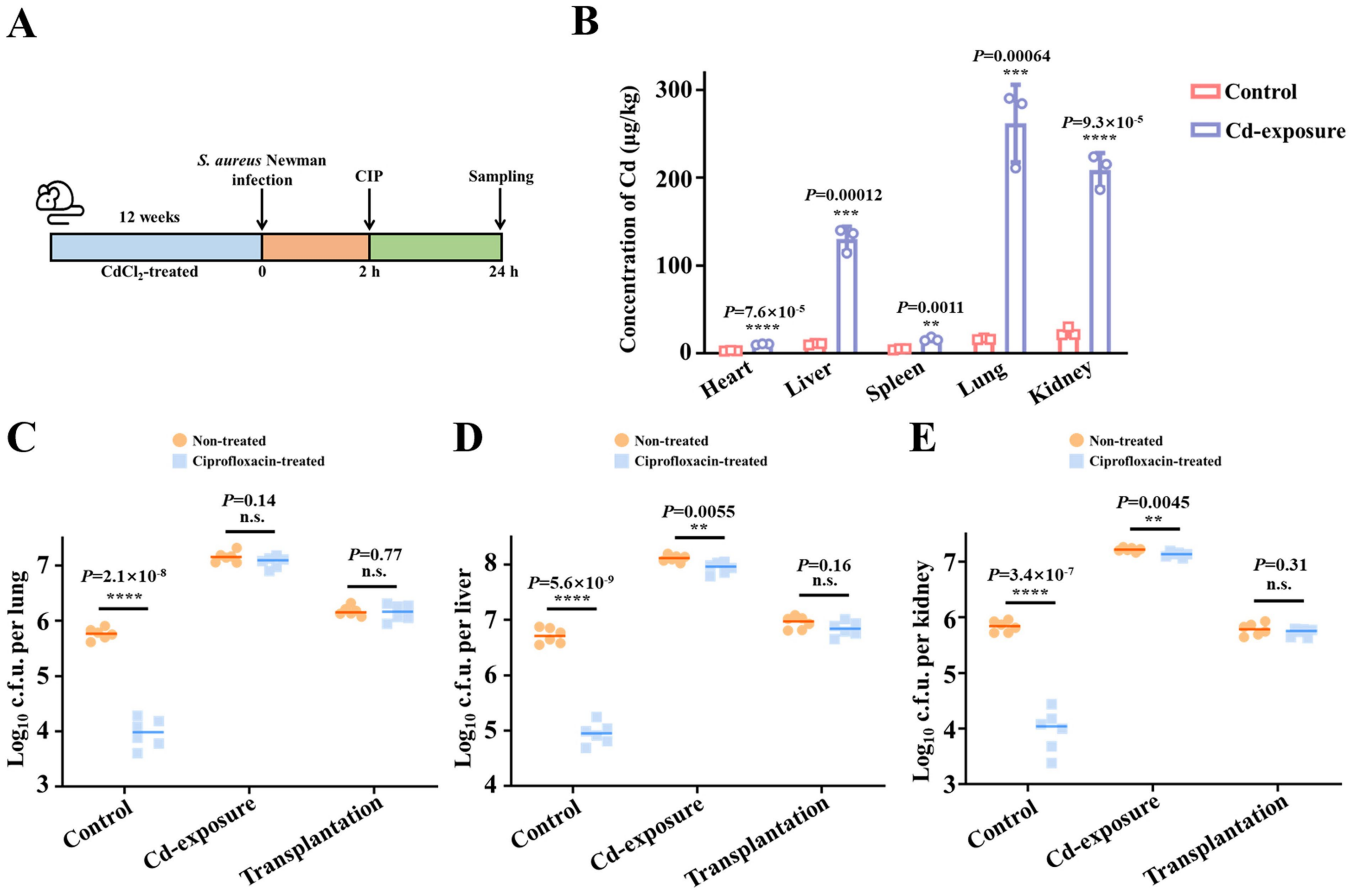
Chronic cadmium exposure decreases the bacterial clearance efficacy of antibiotics in mice models. **(A)** Schematic representation of the experimental protocol depicting the assessment of antibiotic efficacy against *S. aureus* Newman after chronic cadmium exposure of 12 weeks. **(B)** The content change of cadmium in mice viscera after exposure to treatment of CdCl_2_ for a duration of 12 weeks. *n* = 4 biologically independent animals per group. **(C–E)** The effect of chronic cadmium exposure on ciprofloxacin killing against *S. aureus* Newman in mice lungs **(C)**, livers **(D)**, and kidneys **(E)**. Microbiota transplantation from Cd-exposure group was conducted to demonstrate the correlation between impaired antibiotic efficacy and gut microbiota alteration. *n* = 6 biologically independent animals per group. Data are shown in median values. **p < 0.01, ***p < 0.001, ****p < 0.0001. n.s. represents not significant.

To investigate the relationship between low-dose chronic cadmium exposure and antibiotic efficacy *in vivo*, we established a cadmium exposure mouse model by adding 1 mg/L CdCl_2_ to the drinking water. Mice in both the control and cadmium (Cd)-exposure groups were administered ciprofloxacin 2 h following infection with *Staphylococcus aureus* (*S. aureus*) Newman, while the untreated group received phosphate-buffered saline (PBS). Twenty-four-hour post-infection, lungs, livers and kidneys were harvested for bacterial load assessment (Figure 1A). After 12 weeks of exposure, cadmium accumulation in various organs was quantified using inductively coupled plasma mass spectrometry (ICP-MS). As depicted in Figure 1B, cadmium concentrations significantly increased in all tested organs, with the highest accumulation observed in the lungs, livers and kidneys, showing increases of 16.1, 12.3, and 8.7-fold, respectively.

Following the successful establishment of the cadmium exposure mouse model, we assessed whether cadmium exposure leads to a reduction in antibiotic efficacy. Prior to evaluating the impact of cadmium exposure on antibiotic efficacy, we observed that *S. aureus* Newman colonization was significantly enhanced under specific heavy metal stress conditions, indicating increased host susceptibility to bacterial infections. Subsequently, we assessed the reduction of bacterial loads in the three organs with and without ciprofloxacin treatment to determine therapeutic efficacy. In the control group, a single dose of ciprofloxacin resulted in an approximately 100-fold reduction in bacterial loads within the organs. In contrast, the Cd-exposure group exhibited minimal reduction following antibiotic administration, suggesting diminished antibiotic efficacy (Figures 1C–E).

### 2.2 Composition alteration of gut microbiota in Cd-exposure mice is crucial for the decreasing antibiotic efficacy

We then aimed to investigate the underlying mechanisms contributing to the observed antibiotic failure in the Cd-exposure physiological environment. Given the established role of contaminated food and water as significant sources of cadmium exposure (Zhao et al., 2025), and recognizing that the gut microbiota serves as the primary interface for orally ingested substances (Zhang et al., 2020), it is highly probable that cadmium exposure significantly alters the composition of the gut microbiota. Previous studies have demonstrated that acute high-dose cadmium exposure in mouse models disrupts beneficial gut microbiota while promoting harmful bacterial growth (Garg et al., 2025; Nehzomi and Shirani, 2025). To assess whether the disruption of gut microbiota by cadmium exposure critically impairs antibiotic efficacy, a microbiota transplantation experiment was conducted as previously described (Tan et al., 2022).

Initially, the native gut microbiota of the transplantation group mice was depleted by administering a cocktail of antibiotics in their drinking water for 1 week. Fecal samples from Cd-exposure mice were collected, and the extracts were continuously added to the drinking water of the transplantation group for 2 weeks, followed by a return to normal drinking water for the subsequent 9 weeks (Figure 1B). Thereafter, the efficacy of antibiotics was evaluated post-gut microbiota transplantation. In our study, we observed that, in contrast to direct cadmium exposure, microbiota transplantation from Cd-exposure mice did not result in an increased bacterial burden compared to the control group. This suggests that the heightened susceptibility to bacterial infection following cadmium exposure is not attributable to gut microbiota dysbiosis. However, similar to the Cd-exposure mice, a significant reduction in antibiotic efficacy was observed post-microbiota transplantation (Figures 1C–E). These findings led us to hypothesize that cadmium exposure alters the gut microbiota composition, resulting in significant changes in specific metabolites, which ultimately contribute to antibiotic treatment failure.

**Figure 2:**
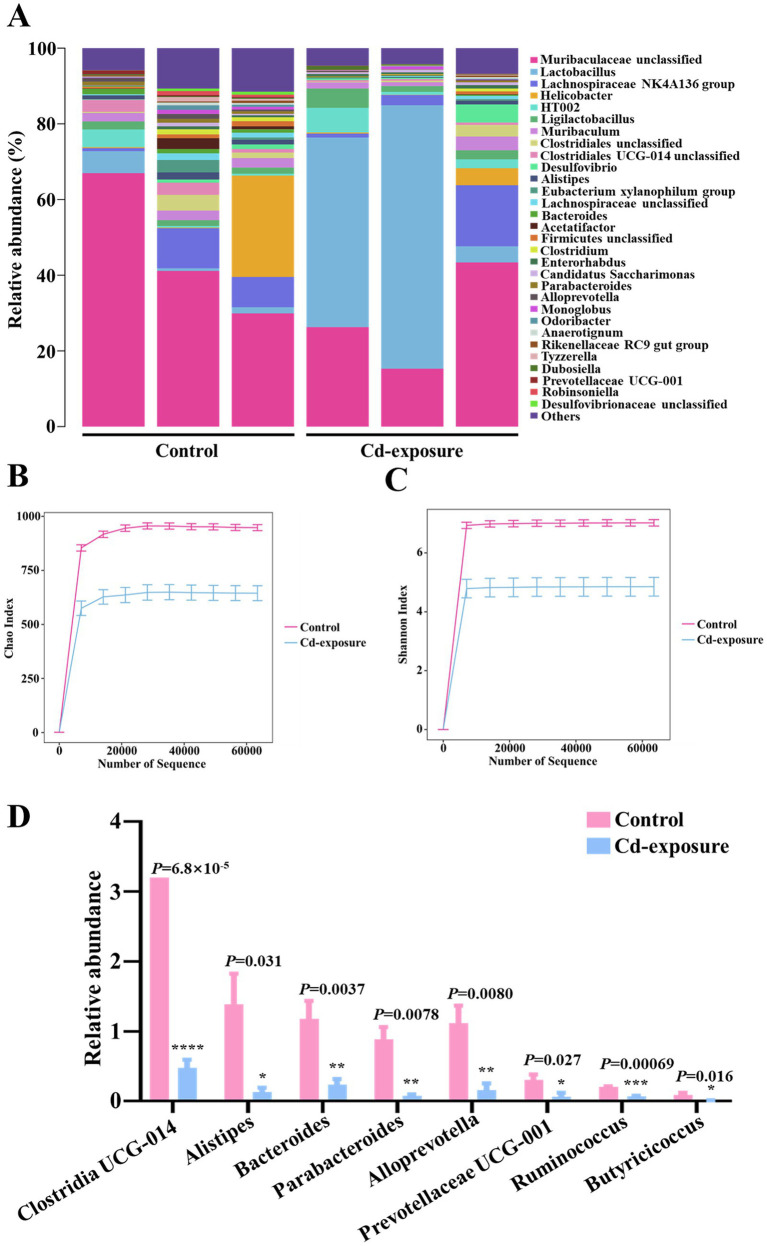
16S rDNA sequencing reveals an alteration in the diversity and composition of gut microbiota in Cd-exposure mice. **(A)** The relative abundance of operational taxonomic units (OTUs) in fecal samples from control or Cd-exposure group mice. *n* = 3 biologically independent animals per group. **(B,C)** Chao richness index **(B)** and Shannon diversity index **(C)** of fecal samples from control or Cd-exposure group mice. *n* = 3 biologically independent animals per group. Data are shown in mean ± s.d. **(D)** The relative abundance of core and beneficial commensals among the top 20 bacterial genera in fecal samples from control or Cd-exposure group mice. *n* = 3 biologically independent animals per group. Data are shown in mean ± s.d.

To investigate the mechanisms by which chronic cadmium exposure diminishes antibiotic efficacy, we conducted 16S rDNA sequencing to assess alterations in gut microbiota composition following cadmium exposure. Our analysis of the microbial community composition revealed a reduction in species diversity within the intestinal flora of Cd-exposure mice (Figure 2A). Additionally, we utilized the Chao and Shannon index to quantitatively compare microbiota diversity between the two groups. In a similar vein, both index exhibited a significant reduction following cadmium exposure (Figures 2B,C). Subsequently, we conducted an in-depth analysis to investigate the differences in the abundance of core and beneficial commensals between the two groups at the genus level. Among the top 20 bacterial genera, the abundance of eight genera, comprising both core and beneficial commensals, was markedly decreased. These included five beneficial gut microbiota genera—Clostridia UCG-014, Alistipes, Prevotellaceae UCG-001, Ruminococcus, and Butyricicoccus—and three core commensal genera—Bacteroides, Parabacteroides, and Alloprevotella (Figure 2D). This suggests that chronic cadmium exposure adversely affects the survival of the aforementioned beneficial bacterial genera. The observed decrease in genera such as Clostridia UCG-014 and Alistipes aligns with previous studies that associated cadmium exposure with gut dysbiosis, highlighting the essential role of these taxa in preserving gut barrier integrity and facilitating short-chain fatty acid production (Garg et al., 2025). Furthermore, the reduction in Butyricicoccus, a recognized butyrate producer, parallels findings from models of dysbiosis induced by heavy metals, where the depletion of butyrate was found to exacerbate metabolic and immune dysfunction (Nehzomi and Shirani, 2025). Collectively, long-term low-dose cadmium exposure in mice resulted in a diminished variability of gut microbiota species diversity, particularly impacting key beneficial bacteria.

### 2.3 DL-mevalonolactone level is reduced with the alteration of gut microbiota

**Figure 3:**
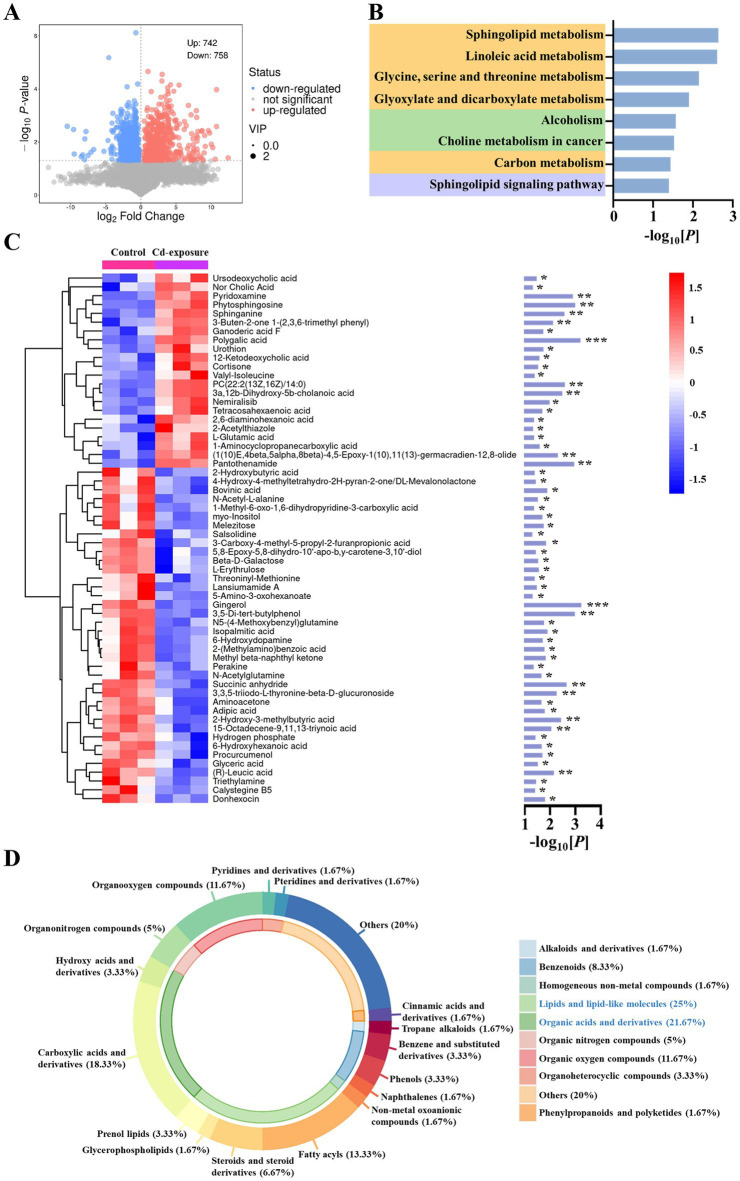
Metabolomics analysis reveals changes of metabolites in Cd-exposure mice. **(A)** The volcano map of differential metabolites for control group v.s. Cd-exposure group. **(B)** KEGG pathway enrichment analysis of significantly regulated metabolites. The corresponding *p* value is shown on the right. Pathways involved in metabolism (yellow), human diseases (green) and environmental information processing (purple) are highlighted. **(C)** Heat-map analysis of the top 60 metabolites in fecal samples from control or Cd-exposure group mice. *n* = 3 biologically independent animals per group. The horizontal coordinates in the figure represent one sample, and the vertical coordinates represent one metabolism. The colors indicate the relative abundance of metabolites, where red represents high content and blue represents low content. The statistical significance (*p* value) of metabolites between two groups is shown on the right. *p < 0.05, **p < 0.01, ***p < 0.001. **(D)** A donut plot illustrating the classification and proportion of differential metabolites. The inner ring of the plot is color-coded according to the Super Class categories, while the outer ring is differentiated by Class. A legend detailing the color keys for the Super Class categories is provided on the right side of the figure. Each segment of the donut plot is distinctly colored to represent a specific classification, and the area of each segment corresponds to the relative proportion of metabolites within the total set of identified differential metabolites.

Given that we have demonstrated the disruption of multiple core and beneficial commensals and the consequent alteration in gut microbiota balance post-cadmium exposure, we further investigated the associated changes in endogenous metabolites. An untargeted metabolomics analysis was performed to investigate changes in metabolite profiles in serum samples from control and cadmium-exposed mice following a 12-week feeding period. The results indicated significant variations in the levels of 1,500 metabolites, with 742 metabolites being upregulated and 758 downregulated (Figure 3A). Subsequent KEGG pathway enrichment analysis of the differentially expressed metabolites revealed a predominant enrichment in pathways related to organic acid and lipid metabolism, including sphingolipid metabolism, linoleic acid metabolism, glycine, serine, and threonine metabolism, glyoxylate and dicarboxylate metabolism, and carbon metabolism (Figure 3B). Additionally, the top 60 metabolites were identified and subjected to clustering analysis, comprising 22 upregulated and 38 downregulated metabolites (Figure 3C). Notably, several key organic-acid-related compounds, such as 2-hydroxybutyric acid (Xie et al., 2025) and DL-mevalonolactone (Yogev et al., 2023), were identified, which have been previously reported as regulatory factors in various diseases and disorders. Moreover, the classification of differential metabolites revealed a significant emphasis on lipids and organic acids, with 25% identified as lipids and lipid-like compounds and 21.67% as organic acids and derivatives (Figure 3D).

**Figure 4:**
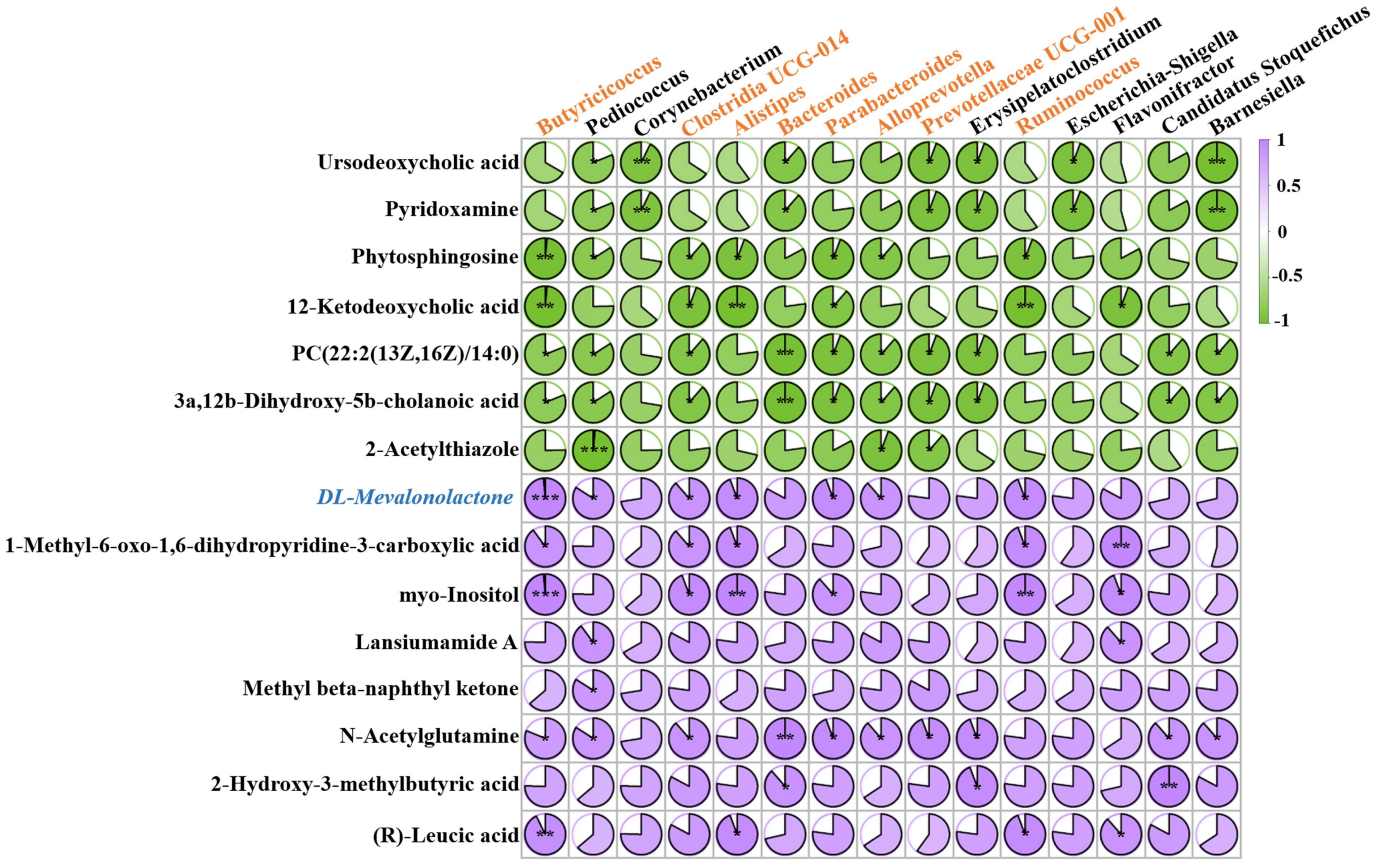
Multi-omics analysis reveals that the circulating level of DL-Mevalonolactone was reduced with the alteration of gut microbiota. The color indicates the correlation coefficients between the top 15 gut microbiota and metabolites. A purple tone represents a positive correlation, and a green tone represents a negative correlation. Darker colors stand for stronger correlations. *p* values for the correlation coefficients were calculated by Pearson correlation coefficient test. *p < 0.05, **p < 0.01, ***p < 0.001.

To investigate the relationship between alterations in gut microbiota composition and circulating metabolite levels, we performed a correlation analysis using the Spearman correlation algorithm. A heatmap illustrating the correlation coefficient matrix between the top 15 gut microbiota and metabolites is presented (Figure 4). As previously noted, the top 15 gut microbiota primarily consisted of various core and beneficial commensals, while the top 15 metabolites were predominantly organic-acid-related compounds and lipid-like molecules. Notably, we observed a significant reduction in the circulating level of DL-mevalonolactone in mice exposed to cadmium compared to control mice. The alteration in serum DL-mevalonolactone content exhibited a strong positive correlation with changes in the abundance of six core and beneficial commensal bacteria, including Butyricicoccus, Clostridia UCG-014, Alistipes, Ruminococcus, Parabacteroides, and Alloprevotella. This suggests that the significant reduction in serum DL-mevalonolactone levels following chronic cadmium exposure is closely associated with alterations in gut microbiota composition, characterized by a notable decrease in the species diversity of core and beneficial commensals. The observed positive correlation between Butyricicoccus and DL-mevalonolactone levels is corroborated by previous research indicating that butyrate-producing bacteria influenced host mevalonate pathway metabolites, which play a critical role in lipid metabolism and immune regulation (LeBlanc et al., 2013). Furthermore, the involvement of Parabacteroides in detoxification pathways (Dubey et al., 2019) may elucidate its association with DL-mevalonolactone, as both are involved in alleviating oxidative stress caused by cadmium exposure. Additionally, myo-inositol and N-acetylglutamine also demonstrated significant correlations with alterations in the gut microbiota, hence the preliminary screening of these candidates was conducted. Despite several metabolites exhibiting noteworthy associations, subsequent functional validation indicated that myo-inositol and N-acetylglutamine did not display measurable biological activity, while the effect of DL-mevalonolactone was remarkable (Supplementary Figure S1). Consequently, our subsequent mechanistic investigations were specifically focused on DL-mevalonolactone. Previous studies have identified DL-mevalonolactone as a *δ*-lactone form of mevalonate, serving as a precursor in the mevalonate pathway (Soto et al., 2011). Oral administration of DL-mevalonolactone has demonstrated efficacy against HMGCR mutation and statin-induced myopathy (Yogev et al., 2023). However, its regulatory effect on bacterial infections has not been documented, and further investigation is required to determine whether the reduced DL-mevalonolactone content is a primary factor contributing to diminished antibiotic efficacy. Furthermore, it remains to be investigated whether this endogenous target metabolite possesses direct antimicrobial activity or contributes to the inhibition of antibiotic tolerance, thereby reducing the formation of bacterial persisters when used in combination with antibiotics. Overall, our findings suggest that administering chronic low-dose cadmium to mice via drinking water may impact the mevalonate pathway and disrupt the biosynthesis of DL-mevalonolactone, potentially indicating an association with diminished antibiotic activity.

### 2.4 DL-mevalonolactone enhances metabolism in *Staphylococcus aureus* and alleviates antibiotic tolerance

**Figure 5:**
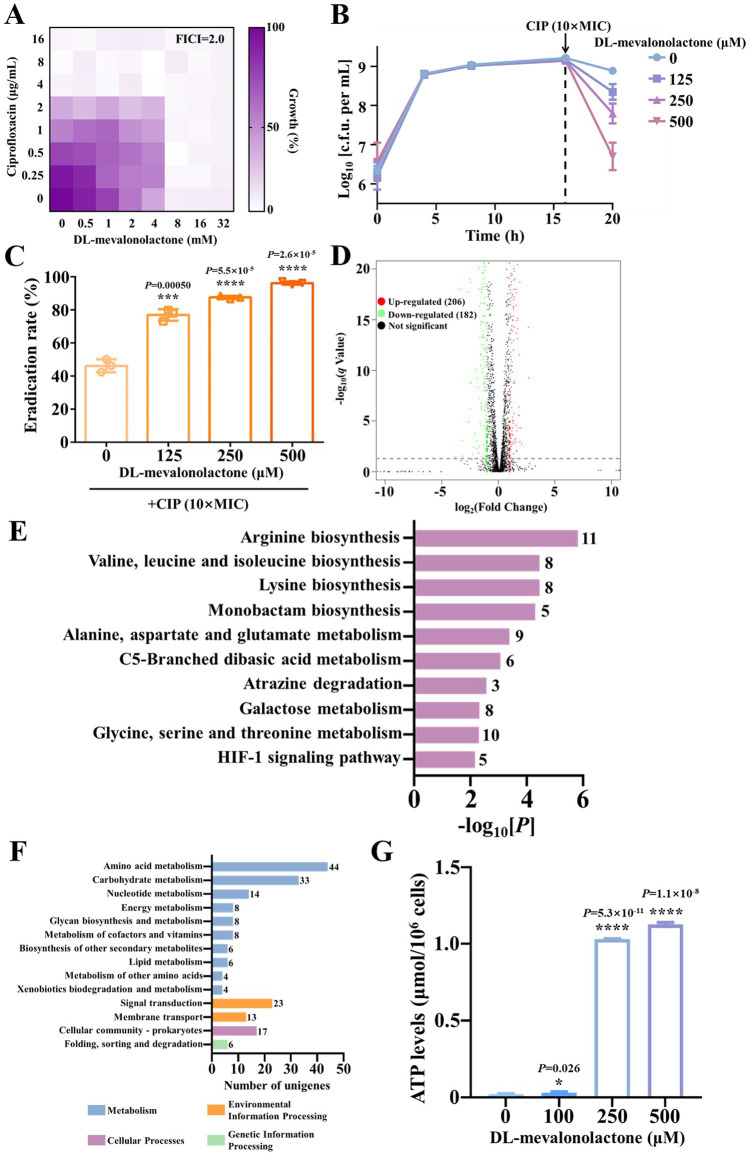
DL-mevalonolactone alleviates antibiotic tolerance potentially through enhancing bacterial metabolism. **(A)** Drug–drug interaction between DL-mevalonolactone and ciprofloxacin against *S. aureus* Newman was evaluated by checkerboard assay. Data represent the mean growth rate of three biological replicates. Fractional inhibitory concentration index (FICI) was calculated as the sum of the two MIC_combination_/MIC_alone_ ratios of two drugs; FICI = FIC_A_ + FIC_B_. Synergism was defined with FICI≤0.5. **(B,C)** Detection of *S. aureus* Newman persisters formation **(B)** and eradication **(C)** in the presence of increasing concentration of DL-mevalonolactone ranging from 0 to 500 μM. Data are shown in mean ± s.d. ***p < 0.001, ****p < 0.0001. **(D)** The volcano map of DEGs in *S. aureus* Newman in the absence and presence of DL-mevalonolactone during 4 h of culture. DEGs were identified with *q* value ≤ 0.05 and |log_2_Fold Change| ≥ 1. Significantly upregulated and downregulated genes are shown in red and green, respectively. **(E,F)** KEGG pathways analysis of DEGs classified at the third-level **(E)** or the second-level **(F)** in *S. aureus* Newman after exposure to DL-mevalonolactone at the time point of 4 h. The name of each KEGG pathway is shown on the left, and the numbers of DEGs enriched in each pathway are shown on the right. **(G)** Intracellular ATP level determination in *S. aureus* Newman persisters after treatment with increasing concentrations of DL-mevalonolactone ranging from 0 to 500 μM. Data are shown in mean ± s.d. *p < 0.05, ****p < 0.0001.

The aforementioned multi-omics analysis suggests that DL-mevalonolactone may play a previously unrecognized role in maintaining antibiotic efficacy. To explore the underlying mechanisms, we conducted further investigations. Initially, *in vitro* minimum inhibitory concentration (MIC) analysis revealed that DL-mevalonolactone alone exhibited weak antibacterial activity, with an MIC value of 8 mM, indicating a minimal direct antibacterial effect. Subsequently, checkerboard assays were conducted to assess the potential synergistic interaction between DL-mevalonolactone and antibiotics. The FIC index indicated that DL-mevalonolactone exhibited minimal *in vitro* synergistic activity with ciprofloxacin against *S. aureus* Newman. Consequently, the potentiation effect observed with bactericidal antibiotics *in vivo* cannot be attributed solely to drug–drug interactions (Figure 5A). We subsequently proposed that the human endogenous metabolite DL-mevalonolactone may induce bacterial activation and inhibit the formation of persisters. Thus, its reduced levels following chronic low-dose cadmium exposure could lead to the emergence of antibiotic tolerance, characterized by phenotypically tolerant bacteria without genetic alterations. To evaluate this hypothesis, we assessed the inhibitory effect of DL-mevalonolactone on the formation of *S. aureus* Newman persisters. Cultures of *S. aureus* Newman were exposed to varying concentrations of DL-mevalonolactone for 16 h, with the highest concentration (500 μM) showing no impact on bacterial growth. Subsequently, ciprofloxacin at 10 times the MIC was administered to eliminate non-persistent bacteria. As anticipated, the rate of persister formation was significantly reduced with the addition of DL-mevalonolactone (Figure 5B). Furthermore, we assessed the eradication effect of DL-mevalonolactone on *S. aureus* Newman persisters. Persister cells were exposed to a combination of ciprofloxacin at 10 times the MIC and varying concentrations of DL-mevalonolactone (ranging from 0 to 500 μM). The eradication rate of persisters was determined using untreated bacteria as a control. Our findings indicate that the addition of DL-mevalonolactone enhances the antibacterial efficacy of ciprofloxacin against *S. aureus* Newman persisters (Figure 5C).

To elucidate the mechanism by which DL-mevalonolactone reduces the formation rate of antibiotic-tolerant bacteria, we conducted a transcriptomic analysis. *S. aureus* Newman, treated with 500 μM DL-mevalonolactone for 12 h, underwent total RNA extraction and sequencing, leading to the identification of differentially expressed genes. The treatment with DL-mevalonolactone resulted in the upregulation of 206 genes and the downregulation of 182 genes (Figure 5D). The expression levels of several genes associated with energy metabolism were notably upregulated, including ATP-dependent ABC transporter genes var*F* and *varG* (log_2_[FC] = 1.54 and 1.45, respectively), as well as genes involved in arginine biosynthesis, namely *argB*/*C*/*F*/*H*/*J* (log_2_[FC] = 1.40, 1.74, 3.09, 1.73, and 1.55, respectively). This suggests that supplementation with DL-mevalonolactone promotes enhanced energy production and arginine metabolism. Conversely, the expression levels of several genes encoding key virulence factors of *S. aureus* were markedly downregulated, including the staphylocoagulase encoding gene *coa*, a multidrug efflux-related gene *norB*, a microbial surface component recognizing adhesive matrix molecule encoding gene *spa*, and a staphylococcal enterotoxin-like toxin X encoding gene *selX* (log_2_[FC] = −1.49, −1.71, −3.63, and −1.98, respectively). These findings indicate that DL-mevalonolactone may also suppress the expression of virulence factors in *S. aureus*. The KEGG enrichment bar chart revealed that differentially expressed genes were predominantly associated with various metabolic pathways, notably including arginine biosynthesis, valine, leucine, and isoleucine biosynthesis, lysine biosynthesis, as well as alanine, aspartate, and glutamate metabolism (Figure 5E). Collectively, these findings suggest that DL-mevalonolactone significantly influenced the metabolism of *S. aureus*, particularly affecting amino acid and energy metabolism (Figure 5F).

Previous studies have indicated that a key factor contributing to antibiotic tolerance is the reduction in bacterial metabolism (Lobritz et al., 2015; Liu et al., 2022). This metabolic deceleration enables bacteria to enter a dormant state, rendering them less vulnerable to antibiotics, which typically target active cellular processes. Given that DL-mevalonolactone supplementation enhances *S. aureus* metabolism, we hypothesize that the reduction in DL-mevalonolactone levels due to chronic low-dose cadmium exposure diminishes bacterial metabolism and energy production, thereby resulting in decreased antibiotic susceptibility. To validate our hypothesis, we assessed alterations in bacterial intracellular ATP levels following treatment with a combination of ciprofloxacin and increasing concentrations of DL-mevalonolactone. Consistent with our expectations, the addition of DL-mevalonolactone significantly enhanced ATP production (Figure 5G), indicating that DL-mevalonolactone may facilitate the transition of dormant bacteria to a metabolically active state by promoting ATP synthesis. This mechanism of metabolic reactivation parallels the effects observed with other small-molecule adjuvants, such as 2-hydroxybutyrate, which also restored bacterial susceptibility by augmenting TCA cycle activity and ATP production (Xie et al., 2025). The association between metabolic activation and the reversal of antibiotic tolerance is further corroborated by studies indicating that the replenishment of essential metabolic intermediates could counteract bacterial dormancy in persistent infections (Lobritz et al., 2015).

## 3 Discussion

Antibiotic tolerance is increasingly recognized as a critical factor in the failure of antibiotic treatments, intricately linked to the specific physiological environments inhabited by bacteria. A primary mechanism underlying antibiotic tolerance is the ability of bacteria to enter a dormant state, characterized by substantially reduced metabolic activity (Pu et al., 2017). This dormancy enables bacteria to withstand the effects of antibiotics, which typically target actively growing cells (Pu et al., 2019). The starvation-signaling stringent response (SR) plays a crucial role in this process by reducing oxidative stress levels in bacterial cells, thereby enhancing their survival during antibiotic treatment (Schofield et al., 2018). The disruption of this protective mechanism has been demonstrated to markedly increase the susceptibility of biofilms to various classes of antibiotics, thereby enhancing the efficacy of antibiotic treatments in experimental infections. Nonetheless, the relationship between environmental exposures, such as chronic cadmium exposure, and the emergence of antibiotic tolerance has not been explored prior to this study.

Our experimental findings indicated that chronic low-dose cadmium exposure facilitated the development of antibiotic tolerance by altering the composition of gut microbiota. This alteration was predominantly characterized by a significant decrease in the relative abundance of core and beneficial commensals, alongside a reduction in circulating levels of DL-mevalonolactone. Correlation analysis results suggested that the decline in the abundance of Butyricicoccus is primarily responsible for the reduced level of DL-mevalonolactone. Furthermore, changes in the abundance of five additional core and beneficial commensals, including Clostridia UCG-014, Alistipes, Ruminococcus, Parabacteroides, and Alloprevotella, were found to be correlated with DL-mevalonolactone content. Notably, *Parabacteroides distasonis* has been documented to enhance gut health and facilitate the excretion of toxic substances such as cadmium in mice exposed to this metal, underscoring its potential role in maintaining metabolic equilibrium (Dubey et al., 2019).

DL-mevalonolactone is a metabolite of considerable physiological importance within the mevalonate pathway, functioning as a crucial intermediate in isoprenoid biosynthesis. The enzymatic conversion of mevalonate to DL-mevalonolactone is well-documented in eukaryotic systems, particularly regarding its regulatory functions in cholesterol biosynthesis and protein prenylation (Soto et al., 2011). In the gut environment, DL-mevalonolactone pools are likely influenced by both hepatic production in the host and potential microbial contributions, although the complete elucidation of bacterial biosynthetic pathways remains an area requiring further investigation (LeBlanc et al., 2013). The physiological significance of this metabolite is highlighted by its consistent detection in systemic circulation through validated liquid chromatography–tandem mass spectrometry (LC–MS/MS) methodologies (Saini et al., 2006).

Our study demonstrated that the reduction in DL-mevalonolactone content, attributed to alterations in gut microbiota composition, significantly contributed to the failure of antibiotic treatments following chronic cadmium exposure. This finding suggests potential clinical applications for reversing antibiotic tolerance induced by cadmium exposure. The oral administration of DL-mevalonolactone has been investigated in various contexts. For instance, in a study involving a patient with a mutation in HMG-CoA reductase, oral administration of mevalonolactone was found to alleviate symptoms of myopathy (Yogev et al., 2023), indicating its potential as a therapeutic agent for managing statin-related muscle issues. Additional research has been conducted to explore the pharmacokinetics of mevalonolactone and its physiological effects. For example, research investigating the effects of rosuvastatin, a type of statin, revealed that the concurrent administration of mevalonolactone could influence the drug’s effects on lung ischemia–reperfusion injury (Matsuo et al., 2015). Furthermore, dietary isoprenoids that suppress mevalonate, which mimic the action of statins, have been demonstrated to affect osteoclastogenesis and osteoblastogenesis, processes essential for bone health maintenance (Mo et al., 2012). These findings provide crucial evidence supporting the safety of DL-mevalonolactone as oral agents in reducing antibiotic tolerance, although further research is warranted.

In addition to elucidating that DL-mevalonolactone depletion under cadmium exposure was significantly associated with antibiotic tolerance, we further investigated the underlying mechanisms. The transcriptomic evidence showing DL-mevalonolactone-induced upregulation of metabolic genes (including arginine biosynthesis pathways and ABC transporters), coupled with its ability to enhance ATP production and reduce persister formation, strongly suggested this metabolite counteracts antibiotic tolerance by reversing bacterial metabolic dormancy - consistent with established mechanisms of metabolic reactivation in tolerant bacteria (Lobritz et al., 2015; Liu et al., 2022). While these findings clearly position DL-mevalonolactone as a crucial mediator between cadmium exposure and antibiotic failure, the precise molecular targets in *S. aureus* (such as potential interactions with VarF or ArgB) and whether its effects are mediated through canonical mevalonate pathways require further investigation.

Ultimately, chronic cadmium exposure has been identified as a significant factor contributing to reduced antibiotic efficacy. The impact of cadmium exposure on antibiotic tolerance in mice was associated with a decrease in the levels of the endogenous metabolite DL-mevalonolactone, mediated by alterations in gut microbiota. Our study demonstrated that DL-mevalonolactone reactivated dormant bacteria by enhancing ATP production, thereby converting tolerant pathogens into antibiotic-susceptible ones. Nevertheless, it should be noted that this study exclusively used female mice to control for hormonal variability, which limits direct extrapolation to male populations. Given documented sex differences in cadmium toxicokinetics (Vahter et al., 2007) and immune responses (Klein and Flanagan, 2016), future studies should systematically compare both sexes to determine whether the observed cadmium-antibiotic tolerance interactions exhibit sexual dimorphism. Our study elucidates the potential adverse effects of cadmium exposure on the efficacy of antibiotic treatments for bacterial infections. Furthermore, it suggests that metabolites such as DL-mevalonolactone, when used as adjuvants alongside antibiotics, may represent a novel strategy to enhance antibiotic efficacy diminished by antibiotic tolerance.

## 4 Materials and methods

### 4.1 Bacterial strains and reagents

The *S. aureus* Newman strain used in this study was kindly gifted by Prof. Wei Xia (Sun-Yat Sen University). Bacteria were cultured in the tryptic soy broth (TSB) media or on TSB agar plates at 37 °C except as specifically stated. Chemical reagents were purchased from Shanghai Sangon Biotech, MCE or J&K Scientific.

### 4.2 Animal studies and ethical statement

Female C57BL/6 mice (aged 6 weeks, 25–28 g) were supplied from Changzhou Kavens Laboratory Animal Co., Ltd. Female mice were selected based on their increased sensitivity to cadmium toxicity due to sex-specific differences in metal metabolism (Vahter et al., 2007), and more stable group-housing conditions for long-term microbiota studies, avoiding male aggression-related confounders (Van Loo and Baumans, 2003). All animal experiments performed in this study were complied with the Declaration of Helsinki and relevant regulations released by the Ministry of Health and the State Science and Technology Commission, People’s Republic of China. All animal experimental protocols were approved by the Ethics Committee of Wuxi people’s hospital, Jiangsu, China (permission number, DL2023019). The laboratory animal use license was SYXK-2020-0010, certified by the Science and Technology Agency of Jiangsu Province.

Drinking water for control group mice was Nongfu natural mineral water, whereas for Cd-exposure group mice, the drinking water contained 1 mg/L CdCl_2_. Both groups were raised in conventional housing for 12 weeks before infection.

### 4.3 Visceral cadmium content determination

After 12 weeks of modeling, hearts, livers, spleens, lungs and kidneys of both group mice were harvested and weighed. The Cd concentrations of these internal organs were analyzed by ICP-MS after nitrolysis to verify the success of the Cd-exposure model.

### 4.4 Antibiotic susceptibility detection in mice

*Staphylococcus aureus* Newman was cultured overnight at 37 °C and diluted 1/100 into fresh TSB media. The liquid culture was grown at 37 °C with shaking (220 r.p.m.) for about 4 h until the OD_600nm_ reached 0.6–0.8, and then bacteria were harvested and washed twice with ice-cold PBS buffer. Bacteria c.f.u. per milliliter was determined before infection.

To evaluate ciprofloxacin efficacy, control and Cd-exposure group mice were infected intraperitoneally with *S. aureus* Newman at a non-lethal dose (1.0 × 10^7^ c.f.u. per mouse). The infected mice received intraperitoneal rejections with a total dose of 20 mg/kg ciprofloxacin or sterile PBS as control at 2 h post-infection. Animals were euthanized 24 h following infection. The lungs, livers and kidneys of each mouse were aseptically removed, homogenized, serially diluted and plated on TSB plates to determine the bacterial c.f.u. in the absence or presence of ciprofloxacin treatments. Six mice were used for each experimental group.

### 4.5 Gut microbiota transplantation

Before gut microbiota transplantation, the transplantation group mice (*n* = 6 biologically independent mice per group) were initially fed with a cocktail of antibiotics in drinking water for 1 week to clear the original gut microbiota. Approximately 100 mg feces from Cd-exposure group mice were collected and resuspended in 1.5 mL sterile PBS, and then the gut microbiota supernatants were obtained after centrifugation. Supernatants were transplanted into the microbiota-depleted mice by gavage for 2 weeks. After transplantation, mice were fed with a standard diet and regular drinking water as the control group for another 10 weeks. Subsequently, the ciprofloxacin efficacy was evaluated as described above.

### 4.6 16S rDNA sequencing

Fresh fecal samples of control and Cd-exposure group mice were collected after feeding for 12 weeks. DNA was extracted using the CTAB according to manufacturer’s instructions and 16S rDNA sequencing was performed by Shanghai Biotree Biotech. For amplicons of the V3-V4 region, primers were 341F (5′-CCTACGGGNGGCWGCAG-3′) and 805R (5′-GACTACHVGGGTATCTAATCC-3′). For amplicons of the Archae region, primers were F (5′-GYGCASCAGKCGMGAAW-3′) and R (5′-GGACTACHVGGGTWTCTAAT-3′). For amplicons of the V4 region, primers were 515F (5′-GTGYCAGCMGCCGCGGTAA-3′) and 806R (5′-GGACTACHVGGGTWTCTAAT-3′). For amplicons of the V4-V5 region, primers were F (5′-GTGCCAGCMGCCGCGG-3′) and R (5′-CCGTCAATTCMTTTRAGTTT-3′). The amplicon pools were prepared for sequencing and the size and quantity of the amplicon library were assessed on Agilent 2100 Bioanalyzer (Agilent, United States) and with the Library Quantification Kit for Illumina (Kapa Biosciences, Woburn, MA, United States), respectively. The libraries were sequenced using the NovaSeq PE250 platform. High-quality clean tags were obtained by fqtrim (v 0.94) and chimeric sequences were filtered using Vsearch software (v 2.3.4). Alpha diversity was applied in analyzing complexity of species diversity for a sample through 5 indices, including Chao1, Observed species, Goods coverage, Shannon, Simpson, and all these indices were calculated with QIIME2. Beta diversity was calculated by QIIME2, the graphs were generated by R package. Blast was used for sequence alignment, and the feature sequences were annotated with SILVA database for each representative sequence.

### 4.7 Untargeted metabolomics analysis

For metabolites extraction, 100 μL serum sample from control and Cd-exposure group mice after 12 weeks of feeding were mixed with 400 μL extraction solution (MeOH: ACN, 1:1) containing deuterated internal standards. Samples were vortexed for 30 s, sonicated for 10 min at 4 °C and incubated at −40 °C for 1 h to precipitate proteins. After centrifugation (13,800 g for 15 min at 4 °C), the supernatants were obtained for LC–MS/MS analysis.

The metabolite extracts were analyzed by a Vanquish UHPLC system (Thermo Fisher Scientific) with a Waters ACQUITY UPLC BEH Amide (2.1 mm × 50 mm, 1.7 μm) coupled to Orbitrap Exploris 120 mass spectrometer (Thermo Fisher Scientific). Water with 25 mmol/L ammonium acetate and 25 mmol/L ammonia hydroxide (pH = 9.75) (A) and acetonitrile (B) were used as the mobile phase. The auto-sampler temperature was 4 °C, and the injection volume was 2 μL.

The ESI source conditions were set as follows: sheath gas flow rate as 50 Arb, Aux gas flow rate as 15 Arb, capillary temperature 320 °C, full MS resolution as 60,000, MS/MS resolution as 15,000, collision energy: SNCE 20/30/40, spray voltage as 3.8 kV (positive) or −3.4 kV (negative), respectively. The raw data were converted to the mzXML format using ProteoWizard and processed with an in-house program, which was developed using R and based on XCMS, for peak detection, extraction, alignment, and integration. Then an in-house MS2 database (BiotreeDB) was applied in metabolite annotation. The cutoff for annotation was set at 0.3.

### 4.8 Multi-omics correlation analysis

Relative abundance of top 15 differential genus and differentially expressed metabolites were used to calculate correlations via Spearman algorithm. The cor.test function was used to determine whether the correlation coefficient between the two numerical vectors was statistically significant. Matrix of correlation coefficients and matrix of *p* values were obtained, and the correlation heatmap was plotted using R package ggplot.

### 4.9 MIC assays

Monoclonal *S. aureus* Newman activated on TSB agar plate was picked and inoculated into fresh TSB medium for overnight culture at 37 °C. Then the bacterial solution was diluted 100 times into fresh TSB medium and the culture was continued for an addition 2 h until the OD_600nm_ reached 1.0. The 100-fold diluted bacterial solution was mixed with an equal volume of twofold gradient diluted DL-mevalonolactone in TSB medium, and the mixtures were added into a 96-well transparent microplate. After 16 h incubation at 37 °C, the lowest ciprofloxacin concentration that the growth of bacteria was invisible was defined as the MIC value. Each experiment was performed with biological replicates.

### 4.10 Checkerboard assays

Bacterial solution preparation was performed as described in MIC assays. Each drug was serially diluted to 8 concentrations (final concentrations ranged from 0 to 4 × MIC) to create an 8 × 8 matrix. Equal volume of drug dilution was mixed with bacterial suspensions in a 96-well transparent microplate. After incubation for 16 h at 37 °C, the OD_600nm_ value of each well was detected by a multifunctional microplate reader. Three biological replicates were performed and the means were used for calculation. Fractional inhibitory concentration index (FICI) was calculated as the sum of the two MIC_combination_/MIC_alone_ ratios of two drugs; FICI = FIC_A_ + FIC_B_. Synergism was defined with FICI ≤ 0.5.

### 4.11 Detection of *Staphylococcus aureus* Newman persisters formation and eradication

*Staphylococcus aureus* Newman was cultured overnight and diluted 1000 times into fresh TSB medium containing different concentrations of DL-mevalonalactone (final concentrations ranged from 0 to 500 μM). The culture was continued at 37 °C for 16 h, and then 10 × MIC ciprofloxacin was added to kill non-persistent bacteria for another 4 h. Samples were removed, serially diluted and plated onto TSB agar plates to calculate bacteria c.f.u. per ml at each time point as indicated.

To determine the effect of DL-mevalonolactone on the eradication of *S. aureus* Newman persisters, surviving bacteria after treatment with ciprofloxacin for 4 h were collected and resuspended in fresh TSB medium for further incubation. 10 × MIC ciprofloxacin in combination with different concentrations of DL-mevalonolactone (final concentrations ranged from 0 to 500 μM) were added for another 4 h treatment. The initial and remaining bacteria c.f.u. per ml were counted by coating plate method as described above, and the eradication rates were calculated.

### 4.12 Transcriptomic analysis

*Staphylococcus aureus* Newman was grown to logarithmic phase in fresh TSB medium, then 500 μM DL-mevalonolactone were added into the dosing group. After 4 h at 37 °C, bacteria were collected and total RNAs were extracted using a Bacterial RNA Kit (Omega Bio-Tek). The transcriptomic sequencing and data analysis were performed by Shanghai Sangon Biotech. Total RNA of samples were sequenced using Illumina HiseqTM. Raw sequenced reads were evaluated by FastQC, filtered using Trimmomatic to obtain clean data and mapped against the genome of *S. aureus* Newman through Bowtie2. After normalization, differentially expressed genes were identified using DESeq2 with *q* value ≤ 0.05 and |log_2_Fold Change| ≥ 1.

### 4.13 Intracellular ATP level determination

Intracellular ATP levels of *S. aureus* Newman persisters were determined using a ATP Content Assay Kit (Shanghai Sangon Biotech) as manufacturer’s instructions. *S. aureus* Newman persisters were prepared as described above and adjusted to OD_600nm_ = 0.6. After treatment with different concentrations of DL-mevalonolactone (final concentrations ranged from 0 to 500 μM) for 2 h, bacterial precipitates were collected by centrifugation. Lysostaphin was used to lyse *S. aureus* Newman, and supernatants were obtained for intracellular ATP levels determination. Detecting solution was prepared according to instructions and mixed with samples or standard solutions immediately before optical density A1 at 340 nm was determined. After incubation at 25 °C for 3 min, optical density A2 at 340 nm was determined. Total ATP levels (μmol/106 cells) were calculated as 0.125 × ∆A_sample_÷∆A_standard_.

### 4.14 Statistical analysis

For 16S rRNA data analysis, Wilcoxon tests were used for alpha diversity comparisons while PERMANOVA assessed beta diversity differences. Untargeted metabolomics data utilized Wilcoxon rank-sum tests with FDR correction to identify differential metabolites. Transcriptomic analysis of DEGs was performed using DESeq2’s Wald test. And Spearman’s correlation analysis with FDR adjustment was applied for multi-omics integration, with all analyses conducted in R. Other statistical analysis was performed by GraphPad Prism v.9.5.0 for Windows. All data were presented as mean ± s. d. unless indicated. Differences between two groups were evaluated using unpaired two-tailed Student’s *t*-tests and significance was indicated for *p* < 0.05.

## Data Availability

The datasets presented in this study can be found in online repositories. Metabolomics data have been deposited in Mendeley Data (https://doi.org/10.17632/k8t9f6xwzn.1). 2016S rRNA and RNA-sequencing data have been deposited in the National Center for Biotechnology Information (NCBI) Sequence Read Archive (SRA) database (PRJNA1284633 and PRJNA1284986).
