# Supplementary material for: Cadmium’s silent sabotage: unveiling its impact on antibiotic efficacy
Source: Front Microbiol. 2025 Sep 3;16:1658173. doi: 10.3389/fmicb.2025.1658173 (PMC12440957; doi:10.3389/fmicb.2025.1658173)
Supplement: Supplementary file 1 [file Data_Sheet_1.PDF]

Supplementary Material

Cadmium's Silent Sabotage: Unveiling Its Impact on Antibiotic Efficacy

Xuan Tao[1†], Hong Zhou[1†], Haoda Yu[1*], Yan Wu[1*], Tao Bian[1*]

[1] Department of Respiratory Medicine, Wuxi People's Hospital, Wuxi Medical Center, The Affiliated Wuxi People's Hospital of Nanjing Medical University, Nanjing Medical University, Wuxi, Jiangsu, 214023, People's Republic of China.

† These authors contributed equally to this work.

*** Correspondence:**
Tao Bian
btaophd@sina.com

Yan Wu
15861597129@163.com

Haoda Yu
yhd9988@sina.com

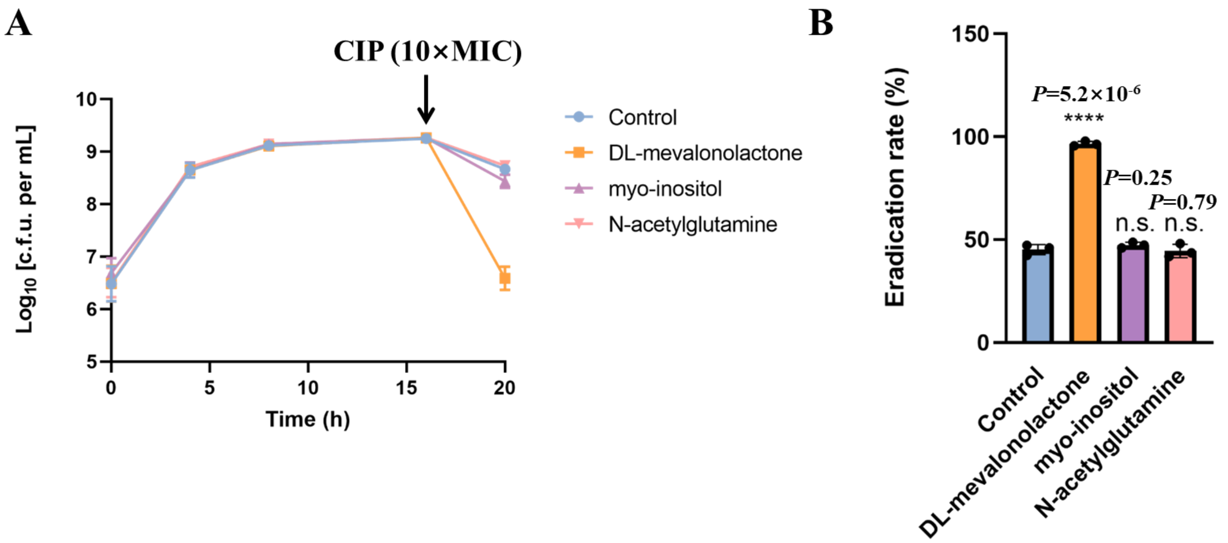

Supplementary Figure 1. Functional validation for candidates through detection of *S. aureus* Newman persisters formation (A) and eradication (B) in the presence of 500 μM DL-mevalonolactone/ myo-inositol/ N-acetylglutamine. Data are shown in mean ± s.d. ****P<0.0001, n.s. represents not significant.

Supplementary Table 1. Exact *P* values of Figure 3B.

KEGG pathway	*P* value
Sphingolipid metabolism	0.0022
Linoleic acid metabolism	0.0024
Glycine, serine and threonine metabolism	0.0071
Glyoxylate and dicarboxylate metabolism	0.012
Alcoholism	0.027
Choline metabolism in cancer	0.029
Carbon metabolism	0.036
Sphingolipid signaling pathway	0.040

Supplementary Table 2. Exact *P* values of Figure 3C.

Metabolites	*P* value
Ursodeoxycholic acid	0.033
Nor Cholic Acid	0.047
Pyridoxamine	0.0012
Phytosphingosine	0.0010
Sphinganine	0.0026
3-Buten-2-one 1-(2,3,6-trimethyl phenyl)	0.0075
Ganoderic acid F	0.018
Polygalic acid	0.00061
Urothion	0.018
12-Ketodeoxycholic acid	0.026
Cortisone	0.029
Valyl-Isoleucine	0.040
PC(22:2(13Z,16Z)/14:0)	0.0026
3a,12b-Dihydroxy-5b-cholanoic acid	0.0032
Nemiralisib	0.010
Tetracosahexaenoic acid	0.020
2,6-diaminohexanoic acid	0.042
2-Acetylthiazole	0.046
L-Glutamic acid	0.042
1-Aminocyclopropanecarboxylic acid	0.026
(1(10)E,4beta,5alpha,8beta)-4,5-Epoxy-1(10),11(13)-germacradien-12,8-olide	0.0048
Pantothenamide	0.0011
2-Hydroxybutyric acid	0.040
4-Hydroxy-4-methyltetrahydro-2H-pyran-2-one/DL-Mevalonolactone	0.036

**Metabolites**	***P* value**
Bovinic acid	0.012
N-Acetyl-L-alanine	0.030
1-Methyl-6-oxo-1,6-dihydropyridine-3-carboxylic acid	0.043
myo-Inositol	0.020
Melezitose	0.018
Salsolidine	0.049
3-Carboxy-4-methyl-5-propyl-2-furanpropionic acid	0.014
5,8-Epoxy-5,8-dihydro-10'-apo-b,y-carotene-3,10'-diol	0.036
Beta-D-Galactose	0.029
L-Erythrulose	0.027
Threoninyl-Methionine	0.040
Lansiumamide A	0.033
5-Amino-3-oxohexanoate	0.048
Gingerol	0.00058
3,5-Di-tert-butylphenol	0.0010
N5-(4-Methoxybenzyl)glutamine	0.017
Isopalmitic acid	0.012
6-Hydroxydopamine	0.019
2-(Methylamino)benzoic acid	0.016
Methyl beta-naphthyl ketone	0.014
Perakine	0.044
N-Acetylglutamine	0.021
Succinic anhydride	0.0022

**Metabolites**	***P* value**
3,3,5-triiodo-L-thyronine-beta-D-glucuronoside	0.0053
Aminoacetone	0.022
Adipic acid	0.016
2-Hydroxy-3-methylbutyric acid	0.0036
15-Octadecene-9,11,13-triynoic acid	0.0087
Hydrogen phosphate	0.038
6-Hydroxyhexanoic acid	0.021
Procurcumenol	0.019
Glyceric acid	0.030
(R)-Leucic acid	0.0069
Triethylamine	0.035
Calystegine B5	0.038
Donhexocin	0.015

Supplementary Table 3. Exact *P* values of Figure 4.

	But.	*Ped.*	*Cor.*	*Clos_UCG.*	*Ali.*	*Bac.*	*Par.*	*Allo.*	*Prev_UCG.*	*Ery.*	*Rum.*	*E-S.*	*Fla.*	*Ca._Stoq.*	*Bar.*
**M1**	0.15	0.049	0.0076	0.17	0.24	0.033	0.10	0.058	0.016	0.016	0.24	0.016	0.29	0.058	0.0028
**M2**	0.15	0.049	0.0076	0.17	0.24	0.033	0.10	0.058	0.016	0.016	0.24	0.016	0.29	0.058	0.0028
**M3**	0.00031	0.036	0.10	0.033	0.016	0.058	0.016	0.033	0.10	0.10	0.016	0.10	0.058	0.13	0.13
**M4**	0.00031	0.083	0.17	0.016	0.0028	0.10	0.033	0.10	0.17	0.13	0.0028	0.17	0.016	0.10	0.24
**M5**	0.049	0.036	0.10	0.033	0.10	0.0028	0.016	0.033	0.016	0.016	0.10	0.10	0.17	0.033	0.033
**M6**	0.049	0.036	0.10	0.033	0.10	0.0028	0.016	0.033	0.016	0.016	0.10	0.10	0.17	0.033	0.033
**M7**	0.083	0.00031	0.083	0.10	0.13	0.10	0.058	0.016	0.033	0.17	0.13	0.13	0.10	0.24	0.10
**M8**	0.00031	0.036	0.10	0.033	0.016	0.058	0.016	0.033	0.10	0.10	0.016	0.10	0.058	0.13	0.13
**M9**	0.015	0.083	0.17	0.033	0.016	0.17	0.10	0.13	0.24	0.24	0.016	0.24	0.0028	0.13	0.29
**M10**	0.00031	0.083	0.17	0.016	0.0028	0.10	0.033	0.10	0.17	0.13	0.0028	0.17	0.016	0.10	0.24
**M11**	0.083	0.015	0.15	0.058	0.10	0.13	0.10	0.058	0.10	0.24	0.10	0.24	0.033	0.17	0.17
**M12**	0.17	0.036	0.10	0.10	0.17	0.10	0.13	0.10	0.058	0.13	0.17	0.17	0.10	0.10	0.10
**M13**	0.049	0.036	0.10	0.033	0.10	0.0028	0.016	0.033	0.016	0.016	0.10	0.10	0.17	0.033	0.033
**M14**	0.083	0.17	0.083	0.058	0.10	0.033	0.10	0.17	0.10	0.016	0.10	0.10	0.13	0.0028	0.058
**M15**	0.0076	0.17	0.083	0.058	0.016	0.13	0.10	0.17	0.24	0.10	0.016	0.10	0.033	0.058	0.17

***** Microbial genera are abbreviated as follows: *Butyricicoccus* (*But.*), *Pediococcus* (*Ped.*), *Corynebacterium* (*Cor.*), *etc.*; metabolites M1–M15 correspond to those labeled in Figure 4 (in order of presentation).

Supplementary Table 4. Exact *P* values of Figure 5E.

KEGG pathway	*P* value
Arginine biosynthesis	1.5×10^{-6}
Valine, leucine and isoleucine biosynthesis	3.5×10^{-5}
Lysine biosynthesis	3.5×10^{-5}
Monobactam biosynthesis	4.9×10^{-5}
Alanine, aspartate and glutamate metabolism	0.00040
C5-Branched dibasic acid metabolism	0.00085
Atrazine degradation	0.0026
Galactose metabolism	0.0047
Glycine, serine and threonine metabolism	0.0049
HIF-1 signaling pathway	0.0068
